# The Role of Extracellular DNA in Microbial Attachment to Oxidized Silicon Surfaces in the Presence of Ca^2+^ and Na^+^

**DOI:** 10.1021/acs.langmuir.1c01410

**Published:** 2021-08-04

**Authors:** Ana L. Morales-García, Rachel Walton, Jamie T. Blakeman, Steven A. Banwart, John H. Harding, Mark Geoghegan, Colin L. Freeman, Stephen A. Rolfe

**Affiliations:** # Department of Physics and Astronomy, The University of Sheffield, Hounsfield Road, Sheffield S3 7RH, U.K.; ‡ Department of Animal and Plant Sciences, The University of Sheffield, Western Bank, Sheffield S10 2TN, U.K.; ø Department of Civil and Structural Engineering, The University of Sheffield, Sheffield S3 7HQ, U.K.; † Department of Materials Science and Engineering, The University of Sheffield, Mappin Street, Sheffield S1 3JD, U.K.

## Abstract

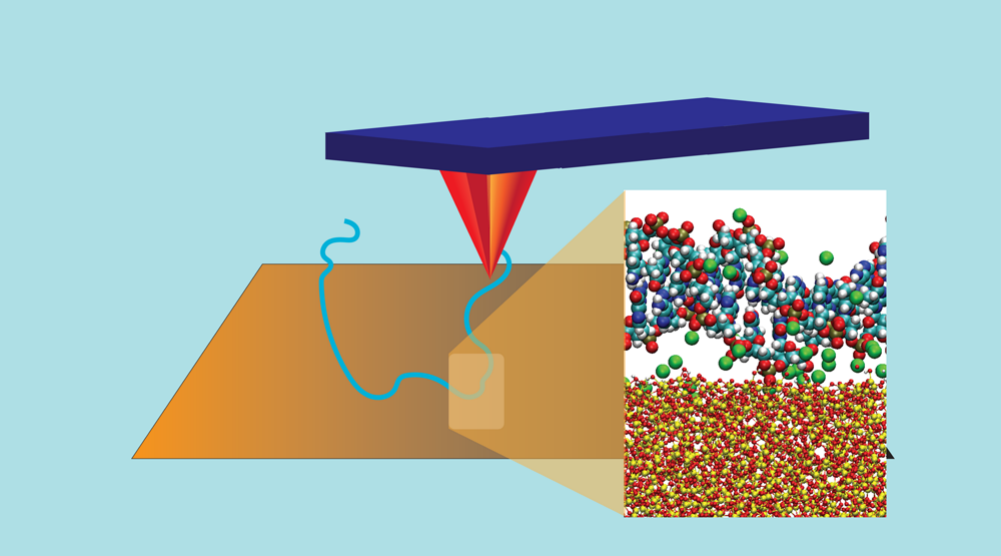

Attachment assays of a *Pseudomonas* isolate to fused silica slides showed that treatment with DNaseI significantly inhibited cellular adsorption, which was restored upon DNA treatment. These assays confirmed the important role of extracellular DNA (eDNA) adsorption to a surface. To investigate the eDNA adsorption mechanism, single-molecule force spectroscopy (SMFS) was used to measure the adsorption of eDNA to silicon surfaces in the presence of different concentrations of sodium and calcium ions. SMFS reveals that the work of adhesion required to remove calcium-bound eDNA from the silicon oxide surface is substantially greater than that for sodium. Molecular dynamics simulations were also performed, and here, it was shown that the energy gain in eDNA adsorption to a silicon oxide surface in the presence of calcium ions is small and much less than that in the presence of sodium. The simulations show that the length scales involved in eDNA adsorption are less in the presence of sodium ions than those in the presence of calcium. In the presence of calcium, eDNA is pushed above the surface cations, whereas in the presence of sodium ions, short-range interactions with the surface dominate. Moreover, SMFS data show that increasing [Ca^2+^] from 1 to 10 mM increases the adsorption of the cations to the silicon oxide surface and consequently enhances the Stern layer, which in turn increases the length scale associated with eDNA adsorption.

## Introduction

Biofilms are widespread in the natural environment with most microbial cells attached to a surface rather than existing in the planktonic form. Adherence to surfaces is beneficial to microbes for many reasons including access to nutrients and redox acceptors in the substratum, facilitating the development of protective biofilms and maintenance of cells in favorable environmental conditions. However, biofilms can pose serious problems such as the biofouling of water supply systems, formation of antibiotic-resistant infections, and attachment to medical implants.^1^ Understanding the processes that govern microbial cell attachment is therefore of great importance.

Biofilms form through cell–surface and cell–cell interactions, with extracellular polymeric substances (EPS) involved in the adhesion processes. Many biopolymers can act as a “glue” including polysaccharides, proteins, phospholipids, and extracellular DNA (eDNA).^2^ Extracellular DNA plays a key role in the structure of many biofilms.^3,4^ In one study of freshwater bacteria, 25 out of 110 isolates produced large amounts of eDNA.^5^ For example, *Pseudomonas* sp. FW1 accumulated eDNA in the stationary phase where it formed a filamentous mesh, linking cells. Similar filamentous structures were seen transiently in a *Reinheimera* sp. F8 isolate, but eDNA production continued, with cells producing large amounts of eDNA (33 μg mL^–1^) to form a confluent mesh. Likewise, meshes of eDNA have also been reported in *Staphylococcus aureus*.^6,7^ In contrast, in *Pseudomonas aeruginosa*, eDNA is associated with specific biofilm structures such as the stalks within mushroom-shaped towers,^8^ during trail formation in biofilm expansion,^9^ and with type IV pili.^10^ The roles played by eDNA in biofilms are varied and can depend on interactions with other EPS components.

The timing and extent of eDNA production are related to the sensitivity of attached cells and biofilms to DNaseI treatment. It has been shown that *P. aeruginosa* biofilm formation was reduced by DNaseI,^4^ which has led to therapies for patients with cystic fibrosis suffering from infections with this organism. Treatment with DNaseI has also been shown to reduce initial cell adhesion to glass surfaces, although the impact on biofilm formation depended upon the species and time of application.^5^ Another report demonstrated that early-stage biofilm formation of *Rhodococcus ruber* (C208) on polyethylene was reduced by DNaseI but that mature biofilms were unaffected.^11^ Considerable variation is seen even within a single species. Some studies report that eDNA in *S. aureus* is involved in early cell attachment, whereas others report greater importance in later stages of biofilm formation.^7^ Extracellular DNA may be released from cells by controlled autolysis^12,13^ or by lysis-independent vesicular routes.^4,14^ Biofilms of *Micrococcus luteus* have been shown to be disrupted significantly more by DNaseI treatment than by enzymes that attack other biofilm components,^15^ although a subsequent study of *P. aeruginosa* biofilms showed that the nature of the polysaccharides in the EPS matrix determined the efficacy of DNaseI.^16^

There is also variation in the reported length of eDNA molecules required for adhesion. In *Listeria monocytogenes*, DNA longer than 500 bp was required for attachment,^17^ while long DNA molecules adsorbed more readily to *Escherichia coli* cells than shorter molecules.^18^ Extracellular DNA facilitated bacterial aggregation in *Streptococcus mutans*, *P. aeruginosa*, and *Staphylococcus epidermidis* at 4 × 10^–9^ to 6 × 10^–9^ μg of DNA per bacterium. Extended DLVO (Derjaguin, Landau, Verwey, and Overbeek) calculations implicated attractive Lifshitz–van der Waals and acid–base interactions, while atomic force microscopy (AFM) showed that acid–base interactions enhanced DNA-mediated aggregation. The impact of eDNA on the strength of interaction was small (1–2 nN), but eDNA greatly extended the range over which such interactions occurred, as would be expected for a long biopolymer.

Divalent cations play an important role in macromolecule interactions as they can form bridges between negatively charged macromolecules and substrates. For example, Ca^2+^ enhances adhesion of *P. aeruginosa* to alginate films,^19^ decreases the separation distance between *P. fluorescens* and glass,^20^ and enhances biofilm formation of *Xylella fastidiosa* on polystyrene.^21^ Ca^2+^ binds to DNA^22^ and enhances eDNA-mediated cell aggregation in species including *P. aeruginosa*, *Enterococcus faecalis*, and *Aeromonas hydrophila*.^23^ Indirect effects may also occur: the interaction of charged eDNA with other charged molecules on the cell surface may lead to an increase in overall cell hydrophobicity as polar units become saturated.

DNA adhesion to silica surfaces has been widely studied. For example, the DNA structure (super-coiled or linear) had no effect on DNA binding to sand particles but increasing the concentration of sodium in the solution increased the quantity bound.^24^ In low ionic strength solutions, only weak interactions between the silica surface and biopolymer analogues of DNA were found using AFM. The role of the solution ions has been explored further, demonstrating that different ion types (e.g., chaotropic or kosmotropic salts) can control the interactions.^25^ On mica, which has a similar charge to silica, polymer binding depends on the concentration and type of monovalent ion present, potentially leading to repulsion between the surface and polymer.^26,27^ Scanning force microscopy experiments have also shown (for divalent and trivalent cations) that the adsorption is largely electrostatic and is impeded on silylated mica.^28^ The quartz crystal microbalance (QCM) is a versatile technique for probing macromolecular adsorption,^29,30^ with studies indicating different binding characteristics between monovalent and divalent cations, with divalent cations generating a much stiffer interface between the DNA and silica.^31,32^ These results are surface-dependent, with the opposite possible (i.e., a stiffer interface for sodium cations than calcium) even for surfaces of the same charge.^31^ Cations are presumed to neutralize the negatively charged DNA molecule, thus reducing the electrostatic repulsion between it and the negatively charged silica.^33^

Molecular interactions with silicate surfaces have been extensively modeled with atomic-scale computer simulations.^34−36^ Several different force fields have been suggested for tackling the water–SiO_2_ interface.^37^ The simulations suggested that molecule–surface interactions take place through charged residues and combinations of weaker hydrophobic interactions leading to behavior influenced by the peptide sequence.^38^ Amorphous silica presents additional challenges for simulations due to the irregular, non-periodic structure and large surface charges present at pH 7. DNA binding simulations to this surface have previously been attempted,^39^ mainly to examine the differences between single- and double-stranded DNA. These simulations demonstrated that direct phosphate–silanol interactions occurred at the surface along with hydrophobic interactions between the DNA bases and sections of the surface with near neutral charge. They highlighted the greater flexibility of single-stranded than double-stranded DNA, which maximized the interactions with the surface and therefore gave stronger adsorption.

Many studies have tackled individual length scales of biofilm formation, but gaining a mechanistic understanding is challenging as the systems must be studied across many scales: from the bulk behavior of microbial cells down to the interactions of individual macromolecules to the atom-level interactions that ultimately govern adhesion. In an earlier work on a strain of *Pseudomonas* that uses eDNA to adhere to substrata and form biofilms, it was observed that the eDNA is tightly associated with the cells and does not form an extensive mesh, thus simplifying the study of eDNA-mediated attachment processes.^40^ Here, combined measurements of cell adherence, single-molecule force spectroscopy (SMFS, a technique derived from AFM), and molecular dynamics simulations are used to investigate the mechanistic basis of cell attachment to the substrate in the presence of divalent or monovalent cations, producing an integrated picture of the processes involved. It is shown that divalent cationic environments that lead to stronger interactions between eDNA and silica (as assessed by SMFS) do not necessarily lead to increased cell attachment. Molecular dynamics simulations allow us to resolve this apparent contradiction by showing that Ca^2+^ presents an organized layer through which the eDNA must navigate before forming strong bonds.

## Experimental Section

### Bacterial Culture and DNase Treatment

All bacterial experiments used the *Pseudomonas* strain Pse1.^40,41^ Cells were cultured for 48 h at 20 °C in 100 mL of the LB medium with shaking at 150 rpm until the mid-late exponential phase. DNA in the growth medium was determined by the fluorescence assay (Quantifluor, Promega Ltd., UK).

Cells were centrifuged at 6250*g*_n_ for 20 min and resuspended in one-fifth of the original growth medium. For DNaseI treatment, 10× DNase buffer (Promega) was added to a final concentration of 0.1× and then DNaseI was added at 15 U mL^–1^. The sample was mixed by inversion and incubated at 20 °C for 30 min with occasional gentle mixing. To test the efficiency of the DNaseI treatment, 100 μL of the sample was withdrawn at the start of the assay and bacterial cells were removed by centrifugation at 16,000*g*_n_ for 10 min. The supernatant (19 μL) was mixed with 1 μL of 25 ng μL^–1^ phage lambda DNA (Thermo Fisher Scientific) and incubated as above. Digestion was checked by electrophoresis through 1% agarose gel using undigested lambda DNA as a control. DNase was removed by washing the cells a further two times in a phosphate buffered saline (PBS) medium, which was prepared following a standard protocol (137 mM NaCl, 2.7 mM KCl, 10 mM Na_2_HPO_4_, and 1.8 mM KH_2_PO_4_).

### Attachment Assays

Fused silica slides (UQG Optics, Cambridge, UK) were cleaned by sonication in 1% (w/v) sodium dodecyl sulfate for 30 min and then deionized water for the same time. These surfaces are of sufficient quality to be considered free from impurities and defects. The slides were then washed in acetone and dried in a laminar flow hood. Three circles (1.5 cm in diameter) were drawn on each slide using a PAP pen (Sigma-Aldrich, Gillingham, UK) forming a hydrophobic barrier and covered with a glass coverslip, producing wells containing 150–200 μL of liquid.

Untreated or DNase-treated cells (10 mL) were centrifuged for 10 min at 6250*g*_n_ and gently resuspended in 10 mL of PBS. The cells were recentrifuged and resuspended in PBS (or other media as required) at an optical density at 600 nm (OD600) of 0.61 (corresponding to 1 × 10^9^ cells mL^–1^). The cell suspension (200 μL) was added to an incubation well and incubated at 20 °C for 30 min with gentle orbital mixing at 60 rpm. Care was taken to ensure that the incubation times for all treatments were the same. Each treatment was performed three times. The cell suspension was then gently aspirated from the slide surface, which was then washed by immersion four times in 0.2 μm filtered PBS. Excess PBS was removed using filter paper, and the cells were then covered with a glass coverslip.

### Optical Microscopy

For routine counting of attached cells, slides were viewed on an upright microscope (BX51, Olympus, Southend-on-Sea, UK) using Nomarski optics and a 40× objective. Four images were captured consecutively from each field of view, and three sets of images were taken per well using an Olympus DP71 camera. Most cells were attached firmly and did not move between the four consecutive images, but a few free-floating cells were also present. Automated image analysis was used to count cell numbers within each field of view, discounting unattached cells that did not appear in all four consecutive images. All image analysis was performed using ImagePro Plus (Media Cybernetics, Marlow, UK).

To confirm that the cells were viable, live/dead staining of microbial cells was performed using SYTO9 and propidium iodide using a LIVE/DEAD BacLight bacterial viability kit according to the manufacturer’s instructions (Invitrogen). Cell viability was confirmed by confocal microscopy using a LSM510 Meta confocal microscope (Zeiss, Jena). Cells were stained with 5 μM SYTO9 or 30 μM propidium iodide (Life Technologies).

### DNA Sources

Pse1 genomic DNA and eDNA were isolated from cells in media in late exponential growth. Cells were centrifuged at 6250*g*_n_ for 20 min at 4 °C. The supernatant was stored on ice for eDNA isolation, and the cells were frozen for at least 1 h at −80 °C. Cells were resuspended in 500 μL of 10 mM Tris 1 mM EDTA (ethylenediaminetetraacetic acid) buffer (TE) at pH 8.0, then transferred to a microfuge tube containing 400 μL of 1:1 phenol:chloroform, vortexed vigorously, incubated at −20 °C for 30 min, and then centrifuged at 12,000*g*_n_ for 15 min. The aqueous phase was removed, and the phenol:chloroform extraction was repeated. The DNA in the aqueous phase was precipitated using 50% (v/v) isopropanol and 0.3 M sodium acetate and then resuspended in 50 μL of TE buffer. Extracellular DNA was isolated from the supernatant using ethanol/sodium acetate precipitation with the precipitant from 50 mL of the growth medium resuspended in 500 μL of TE buffer. Both genomic and eDNA were then treated with 1 μg mL^–1^ RNase (Sigma-Aldrich, UK) for 30 min at 60 °C and then 1 μg mL^–1^ proteinase K (Sigma-Aldrich, UK) for 30 min at 37 °C followed by ethanol/sodium acetate precipitation and resuspension in TE buffer. Salmon sperm DNA was obtained from Sigma-Aldrich UK, extracted with phenol:chloroform, and precipitated with sodium acetate/ethanol, as described above before use. Gel electrophoresis showed that both methods produced DNA fragments of ∼20 kbp.

To obtain DNA of defined lengths, Pse1 genomic DNA was double-digested with DraI and HaeI (NEB) for 1.5 h and electrophoresed using 1% agarose gel. Gel slices corresponding to a range of different molar masses were removed and the DNA obtained^42^ in which the gel was sandwiched between two sheets of filter paper (Grade I, Whatman) in a syringe and the fluid containing the DNA pressed out. The DNA was then cleaned using ethanol/sodium acetate precipitation. The molar mass of the extracted DNA fragments was confirmed on 1% agarose gel.

For 20 and 100 bp DNA fragments, random oligonucleotide sequences were designed, which did not self-dimerize (6 bp or more) or form hairpins (4 pm or more) using OligoCalc.^43^ The forward and reverse oligonucleotides were synthesized (Sigma-Genosys) and annealed by heating complementary strands together in a thermal cycler (Techne UK) to 98 °C and then reducing the temperature to ambient at a rate of 1 °C min^–1^. The 20 bp oligomer was 5′-AGCTACGACGAGGACCTGAC-3′ and its complementary strand. The 100 bp oligomer was 5′-ACTGACGAGCCCGGTGTCTCTGCACTTGACCGACCCAA CGCAACGACGGTACTG CGATCACTCGCGTCTGCGATCTACGAGCTACGACGAGGACCTGACG-3′ and its complementary strand.

The 688 bp genomic DNA fragment used in the radiolabeling experiments was obtained by ligating TSP5091 (NEB)-digested Pse1 genomic DNA fragments into EcoRI (NEB)-digested pUC19 and cloning into DH5alpha. Cells were grown overnight on blue-white selective LB plates, and individual colonies were pricked out and PCR-screened for an insert using M13 primers (Sigma-Genosys). Excess primers and nucleotides were then removed, and the PCR products were sequenced by the University of Sheffield Core Genomic Facility.

These sequences were compared to those at the NCBI using BLAST, and PCR primers were designed against a fragment with homology to *Pseudomonas* 23S rRNA DNA. The forward and reverse primers were 5′-AAGCGTGGACGCCAGTTCGC-3′ and 5′-TTCGACGGCCCTTCAGGGGA-3′, respectively (Invitrogen, Life Technologies, UK). Genomic Pse1 DNA as a template was then PCR-radiolabeled using these primers, DreamTaq polymerase (Thermo Fisher Scientific) and 0.02 mM d33P-ATP (PerkinElmer) with 0.04 mM unlabeled dGTC-TP and 0.02 mM unlabeled dATP (Bioline, UK). PCR was performed for 30 cycles of 30 s at 95 °C, 55 °C then 72 °C, and a final extension time of 5 min at 72 °C. An unlabeled genomic fragment of the same length was also amplified using non-labeled dATP. Nucleotides and PCR reagents were removed from the PCR products using a Qiagen Qiaquick PCR purification kit. Labeled DNA (1 μL) yielded 2 × 10^6^ CPM when tested prior to use. The concentration of the radiolabeled DNA was approximately 25 ng μL^–1^ from agarose gel estimation. The unlabeled DNA was measured at 110 ng μL^–1^ using a Nanodrop 8000 (Thermo Fisher Scientific).

A labeled stock DNA sample at approximately 100 ng μL^–1^ was made by mixing 150 μL of the unlabeled DNA with 17 μL of the hot DNA and stored at −20 °C. This stock was further diluted in PBS to give the working concentrations used in an assay of 200 ng mL^–1^.

### DNA-Cell Binding Assays

To determine the kinetics of DNA binding to Pse1 cells, both DNase-treated and untreated cells were centrifuged, washed, and resuspended in PBS to an optical density at 600 nm (OD600) of 0.61 and then further diluted in four volumes of PBS. The DNase activity was confirmed as described earlier using lambda DNA. Reaction volumes (1 mL) containing 500 μL of these cells and 500 μL of the labeled DNA were set up in triplicate to give a final OD600 of 0.061 for the cells and 100 ng mL^–1^ DNA. The reaction volumes were incubated for 1 h at 20 °C with gentle orbital shaking. At 0, 10, 20, 30, and 60 min post-mixing, 150 μL of the reaction mix was removed and centrifuged at 6000*g*_n_ for 15 min, and the supernatants and pellets were separated for analysis.

To quantify the DNA associating with the Pse1 cell surface, DNase-treated and untreated cells were centrifuged, washed, and resuspended in PBS to an OD600 of 0.61 and then further diluted in four volumes of PBS. Volumes of 200 μL of these cells and 200 μL of labeled DNA at concentrations of 0, 2, 20, 200, and 2000 ng mL^–1^ were set up in triplicate to give a final OD600 of 0.061 for the cells and 0, 1, 10, 100, and 1000 ng mL^–1^ DNA. A further set containing 150 μL of cell suspension and 150 μL of 10 μg mL^–1^ DNA was also set up to give a final concentration of 5000 ng mL^–1^. The samples were incubated for 1 h at 20 °C with gentle orbital shaking and then centrifuged at 6000*g*_n_ for 15 min, and the supernatants and pellets were separated for analysis.

To establish whether the DNA–cell surface interactions were reversible and whether DNase treatment was effective, 1 mL of reaction volumes containing either 500 μL of DNase-treated or untreated Pse1 cells at an OD600 of 0.61 and 500 μL of DNA in PBS was set up in triplicate. The cell mixes were divided in two to give 500 μL volumes, and these were incubated for an hour with orbital shaking, following which they were all centrifuged at 6000*g*_n_ for 15 min. The supernatants were removed and mixed in 10 mL of scintillation fluid for counting. To test DNase treatment, 20 μL of 10× DNase buffer, 180 μL of water, and 20 U DNaseI were then added to one of each of the duplicate centrifuged cell samples and the cells were resuspended gently and incubated at 20 °C for 1 h. Cells were spun down as before, and the supernatant and cell pellets were separated for analysis. To establish whether the DNA–cell surface interactions were reversible, the second of each duplicate cell sample was resuspended in 500 μL of PBS. Lambda DNA (5 μL) at 500 ng μL^–1^ (sufficient to cover the cells) was added to each tube and incubated at 20 °C with gentle rocking for 1 h. Cells were spun down as before, and the supernatant and cell pellets were separated for analysis.

In all experiments, the supernatants from the centrifuged cell samples were mixed thoroughly with 10 mL of scintillation fluid (Emulsifier Safe; PerkinElmer). The cell pellets were digested in 150 μL of 10% NaOH overnight before adding 10 mL of scintillation fluid. The digest tubes were rinsed with 150 μL of water, and this was also added to the same scintillation vials to minimize sample loss. Radioactivity was then measured using liquid scintillation counting in a Packard Tri-carb 3100 scintillation counter (Isotech, Chesterfield, UK). Proportional losses of radioactivity due to the half-life of 33P were accounted for in subsequent calculations.

### Single-Molecule Force Spectroscopy

MLCT Si_3_N_4_ probes (Bruker AFM Probes, Camarillo, USA) were cleaned using a homemade oxygen plasma cleaner followed by acid piranha solution^44^ (H_2_SO_4_:H_2_O_2_ (v/v) 7:3) for 1 h and then rinsed with deionized water and ethanol, dried with N_2_ gas, and stored in a sealed container until functionalization. Piranha solution reacts violently with many organic compounds, so extreme care was taken when using it. Metal films were deposited onto the probes using an Auto 306 evaporator (BOC-Edwards, Crawley, UK). A 1 nm adhesive layer of Cr (99.99%, Agar Scientific, England) was deposited followed by a 15 nm layer of Au (99.99% purity, Goodfellow Metals, England).

Cantilevers were incubated with 100 μL of thiolated DNA for 2 min and then rinsed with deionized water. DNA was thiolated at the 5′ end using a 5′ EndTag nucleic acid system (Vector Laboratories, Burlingame, USA). DNA (10 μg mL^–1^ in water) was dephosphorylated with alkaline phosphatase at 37 °C for 30 min. Thiolation was achieved by incubation with adenosine-5′-*O*-(3-thiotriphosphate) and T4 polynucleotide kinase at 37 °C for 30 min. Unincorporated nucleotides were removed using a Mini Quick spin DNA column (Roche Diagnostics, Indianapolis, USA). The DNA concentration was measured using a Nanodrop 8000 spectrophotometer (Thermo Fisher Scientific, Wilmington, USA), diluted to 3 ng mL^–1^, and stored at −18 °C until use.

Silicon wafers of 425 ± 25 μm thickness, with a native oxide superficial layer (Prolog Semicor, Kiev, Ukraine), were cut into rectangles (0.5 × 0.5 cm^2^). The substrates were cleaned using piranha solution for 1 h and then rinsed with copious amounts of deionized water. The wafers were then boiled in water for 1 h with water replaced two to three times during this process. Afterward, the wafers were rinsed with analytical-grade ethanol (Sigma-Aldrich, Dorset, England) and sonicated in HPLC-grade methanol (Fisher, Leicestershire, England). The wafers were kept in a sealed vial containing methanol until use.

Force spectroscopy was performed using an MFP3D (Asylum Research, USA). The microscope was equipped with IgorPro 6.22A software for data acquisition and analysis. Calibration of the cantilevers was done using the thermal tuning method.^45^ Force–distance curves were recorded between the cantilevers and the silicon substrate at a loading rate of approximately 40 nN s^–1^ (spring constant, *k* ≈ 40 pN nm^–1^; force distance, 500 nm; speed, 0.99 μm s^–1^). Sets of 150 force–distance curves were acquired with water and then with 2 mM Na^+^, 20 mM Na^+^, 1 mM Ca^2+^, and 10 mM Ca^2+^. Each curve was inspected for secondary peaks of adhesion and the frequency of multiple events counted. The force of adhesion was determined automatically using IgorPro, as the lowest point in the force axis. The work of adhesion was calculated by integrating the area under the force curve, below the zero-force line.

AFM curves were aligned vertically by calculating the mean force when the tip was distant from the surface, where no interaction was evident, and subtracting this from all values. Horizontal alignments were made by performing a linear regression on the compliance region and extrapolating this to the abscissa. To calculate areas above and below the curves, the mean and standard deviation of force were calculated in the non-interacting region. The onset of positive or negative deviations in force was calculated where four consecutive points varied from the mean force value by more than three standard deviations.

### Computational Methods

Unless otherwise specified, all molecular dynamics simulations were performed using the DL_POLY classic code^46^ within the NVT Nosé–Hoover ensemble (thermostat relaxation time of 0.02 fs) and a time step of 0.5 fs.

The DNA chain was modeled using the general AMBER force field.^47^ The TIP3P force field^48^ was used for the water molecules, and the silica was modeled with the BKS force field.^49^ The interactions between the silica and the water molecules used the parameter set derived by Hassanali et al.^50,51^ This force field set includes a three-body term that prevents the binding of hydroxyl hydrogen to multiple oxygen atoms. This artificial potential model was removed from our simulations, and a slightly increased Si–O–H angular term was used instead (as shown in Table S1a). This prevented any multiple bonding and was considered more accurate for the system. Interactions between the DNA and silica surface were generated by scaling pre-existing inter-atomic potentials.^52^ In this method, the silicon interactions with the oxygen atoms within the DNA used the Buckingham interatomic potentials of the BKS potential but the repulsive A parameter was scaled down with a ratio equal to that of the different charge ratio of the Si–O (DNA) compared to Si–O (silica), P, etc. interactions. These potentials are all listed in Table S1b,c.

The amorphous silica slab was generated from bulk cristobalite formed in an 8 × 10 × 6 supercell to make a cell with a total size of 39.2 × 49.0 × 39.5 Å^3^. This was melted by running successive simulations (starting from 300 K) on the bulk crystal with a time step of 1 fs increasing the temperature by 300 K until a maximum temperature of 5100 K was reached. The slab was then cooled in steps of 100 ps and temperature changes of 1000 K until reaching a final value of 297 K.

The amorphous silica cell was then randomly cleaved in the direction of the cell. The surfaces were separated by approximately 40 Å to generate a cell of 80.0 × 49.0 × 39.5 Å^3^. This surface was then relaxed at 297 K (NVT Hoover relaxation time, 0.1 fs) for 100 ps (when the energy was found to have converged) defining the starting configurations for the cation positions. All four-way rings were converted to silanols, and non-bridging O atoms were converted to geminals where the Si was under-coordinated. These oxygen atoms were then fully hydrolyzed. The surface charge was assumed^51^ to be 0.835*e* nm^–2^ as recorded for pH 7, which equilibrated to a surface charge of −16*e*. Therefore, 16 H were randomly removed from both surfaces to generate the correct charge. The surfaces were then relaxed with the addition of the appropriate number of charge-canceling Na^+^ or Ca^2+^ cations in a vacuum. Finally, 5000 water molecules were added to the simulation and the system was relaxed fully. After this, the water molecules were deleted, and the final silica and cation structure was used for addition of the DNA molecule.

The DNA chain was generated from a random double-chained sequence. This chain was cut to be commensurate with the cristobalite cell, which was found to occur at 11 base pairs in length (sequence TGAACTCGATT). This cell was then placed in a periodic simulation box and solvated with 1500 water molecules and either 38 Na^+^ or 19 Ca^2+^ ions to ensure charge neutrality. The periodicity means that the DNA is infinite, so the chain length cannot be tested. Three different random arrangements of cation positions were attempted with the only restriction being the cations began the simulation within 15 Å of the center of the DNA molecule. Simulations were run at 297 K for 0.1 ns, and the final energies were extracted. The configurations with the lowest energies were then used for subsequent simulations with the silica surfaces.

The final simulation box containing the DNA, silica slab, and charge neutralizing cations was generated by placing the relaxed DNA molecule along with its surrounding cations onto the slab with its neutralizing cations relaxed earlier. The DNA chain was placed approximately parallel to the surface at a range of separations as defined by the separation between the center of mass of the DNA and slab. Simulations were run until the configurational energy had converged (see Figure S1), and then the final energetic averages and structural data were collected over the final 1 ns.

## Results

### Cell Attachment to Fused Silica Surfaces Is Facilitated by Extracellular DNA

**Figure 1:**
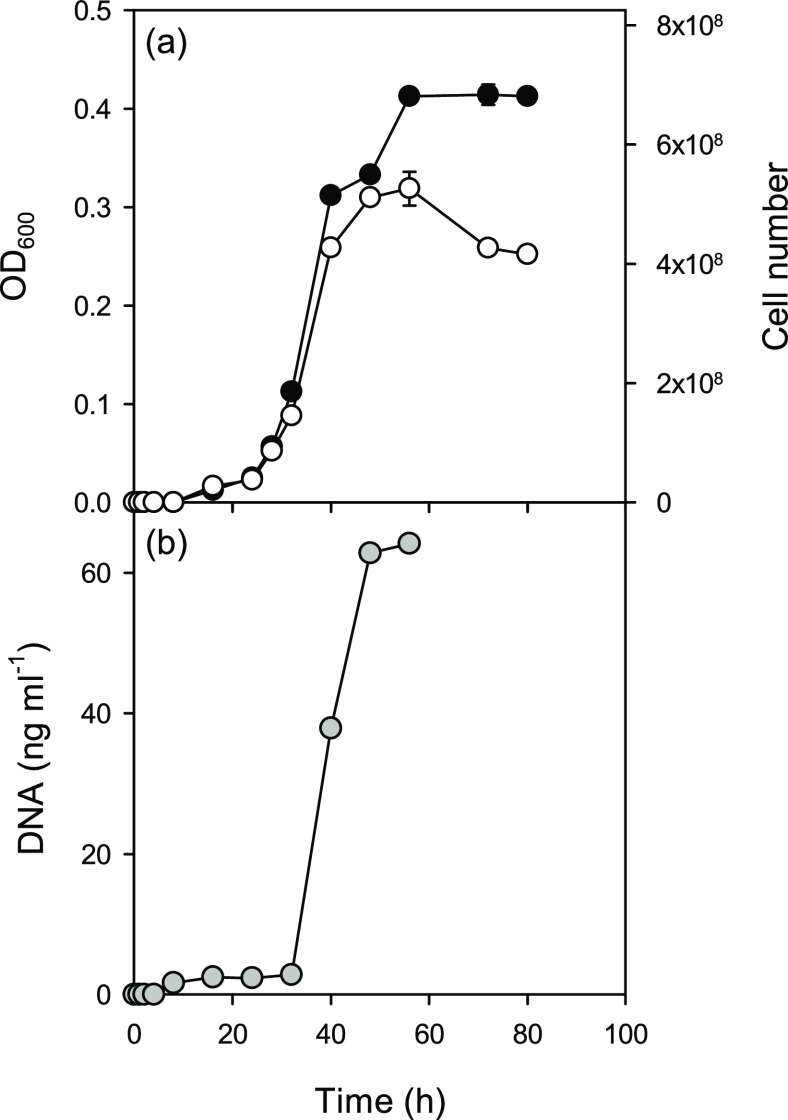
(a) Growth of Pse1 cells in the LB medium as measured by OD600 with (black symbols) and without (open symbols) the addition of DNaseI to the growth medium. The right-hand scale shows calculated cell numbers, and the results are the mean ± SE (standard error) of three replicates. (b) Accumulation of free DNA in the growth medium.

Previous work showed that eDNA facilitated the attachment of Pse1 to surfaces.^40^ Here, initial experiments sought to characterize the properties of eDNA produced by Pse1 and determine the key properties of the eDNA required for cell attachment to fused silica. Figure 1a shows a growth curve of Pse1 in Luria broth (with and without the addition of DNaseI to the medium). Figure 1b shows the accumulation of free DNA in solution. Inoculation of the Luria broth medium with Pse1 resulted in a typical sigmoidal growth curve with an exponential phase 20–40 h after inoculation. When cells were grown without the addition of DNaseI, OD600 dropped at the end of the experiment due to cell aggregation. During the exponential growth phase, double-stranded DNA accumulated in the growth medium reaching a final concentration of 60–70 ng mL^–1^ at the stationary phase (46 h). Gel electrophoresis of the free DNA showed that it was of high molar mass (>20 kbp). Assuming an average genome size^53^ of 6 Mbp for a *Pseudomonas* cell, this amount of DNA represents ∼1% of the cell population. Live/dead staining of the cell population in the stationary phase showed that all cells were viable at this time (detection limit ∼0.01%). Therefore, assuming that this eDNA was released from dead cells, lysis must have been very rapid.

**Figure 2:**
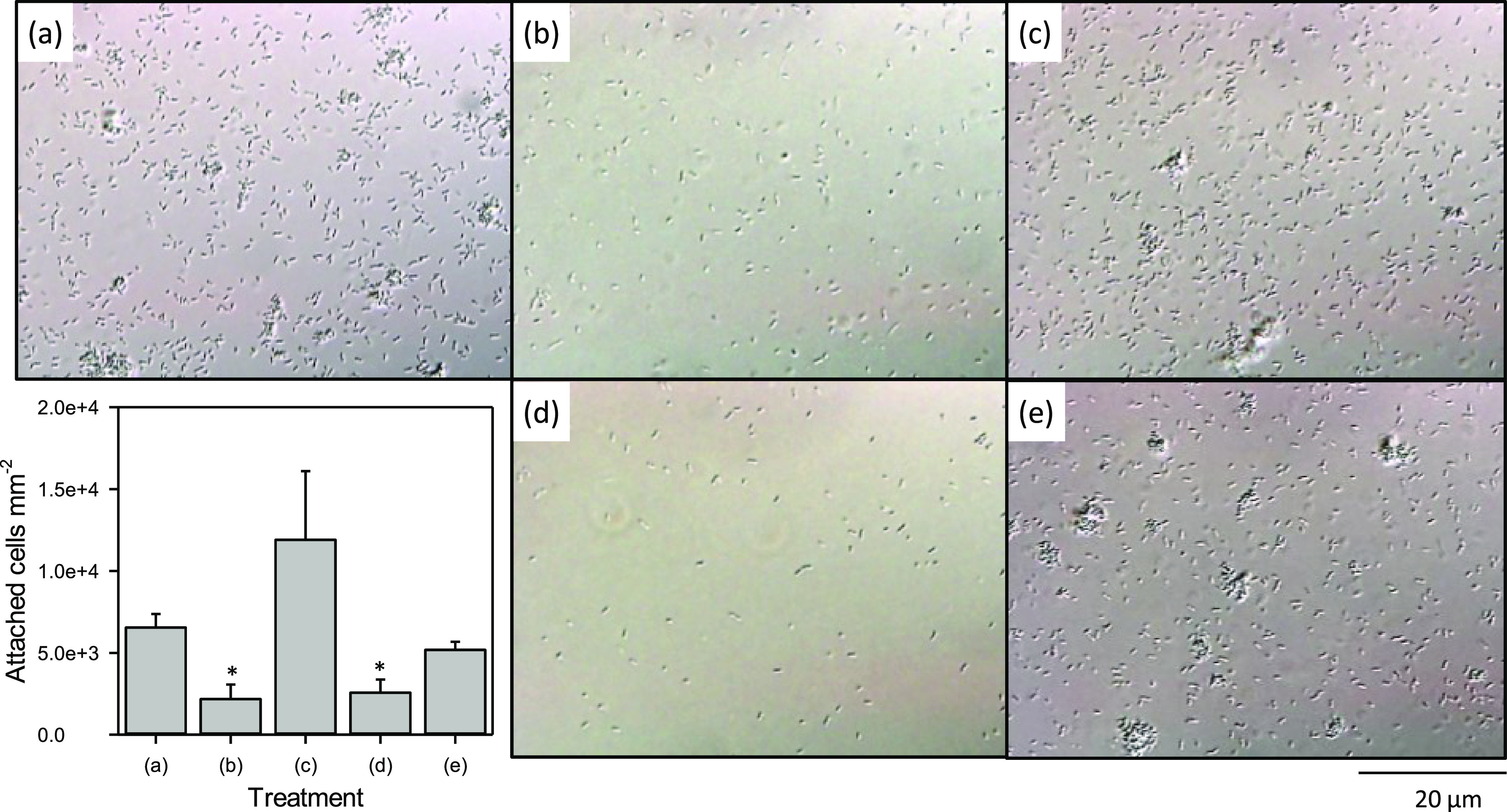
Representative images of cell attachment to fused silica surfaces. (a) Washed cells incubated in PBS, DNase-treated cells incubated in (b) PBS, (c) PBS and 60 ng mL^–1^ eDNA, (d) DNase-treated growth medium, and (e) DNase-treated growth medium and 60 ng mL^–1^ eDNA. One-quarter of a field of view is shown for clarity. The scale bar represents 20 μm. The graph shows mean and SE of attached cells. Asterisks (*) indicate that the value is significantly different from the control (a) (one-way ANOVA of log-transformed cell counts, *n* = 3, *p* < 0.001).

To investigate the role of eDNA in cellular attachment to surfaces, an attachment assay was developed. Bacterial cells were harvested at the late exponential growth phase (46 h), and a proportion was treated with DNaseI to remove eDNA. Cells were then washed twice in phosphate buffered saline (PBS) and resuspended in PBS with additions as required. Cells were then incubated for 30 min on fused silica slides to allow attachment. Unattached cells were removed by washing gently in PBS. Attached cells were visualized using either Nomarski optics or staining with fluorescent dyes followed by fluorescence microscopy. Figure 2a shows washed Pse1 cells attached to the fused silica slide. Numerous individual cells had become attached as well as a few cell aggregates. Treatment of cells with DNaseI (Figure 2b) led to a significant reduction in the number of attached cells with no cell aggregates present. Both cell attachment and the formation of aggregates were restored by the addition of purified eDNA at 60 ng mL^–1^ (Figure 2c). Cell attachment was shown to be dependent on eDNA rather than other factors present in the growth medium because binding was low with the DNaseI-treated growth medium (Figure 2d) and restored by the addition of 60 ng mL^–1^ eDNA (Figure 2e).

To determine whether the source of DNA was important, attachment assays were performed on DNase-treated cells incubated with eDNA extracted from the supernatant, Pse1 genomic DNA, and sheared salmon sperm DNA. All were equally effective at restoring the attachment (Figure S2a). The DNA used in these experiments was 20–25 kbp long. The length of the DNA fragment was not important in determining the Pse1 attachment as binding was restored by fragments ranging from 300 to 10,000 bp (Figure S2b). These experiments were performed at a constant DNA concentration (mass per unit volume), meaning that the charges associated with DNA remained roughly the same, although the number of DNA molecules (moles per unit volume) decreased as the size of the DNA fragments was increased.

### Association of DNA with the Pse1 Cell Surface

To determine how eDNA binds to the surface of Pse1 cells, a fragment of the Pse1 genome was amplified and cloned. Radiolabeled DNA was then prepared by PCR amplification of the cloned fragment with ^33^P-labeled dATP added to the reaction mixture. After purification to remove unbound nucleotides and other PCR reaction components, the radiolabeled product was mixed with unlabeled 688 bp DNA to produce a stock solution of known concentration and specific activity.

To assess the speed with which DNA associated with the cell surface, PBS-washed or DNase-treated Pse1 cells at a concentration of 1 × 10^8^ cells mL^–1^ were mixed with 100 ng mL^–1 33^P-labeled DNA and incubated, with gentle mixing, for periods of up to 60 min. Samples were withdrawn at intervals; the cells and bound eDNA precipitated by centrifugation and the proportion of the radiolabel in the supernatant and cell fraction measured by scintillation counting. At each time point, including a time of 0, ∼30–35% of the available DNA was found in the cell pellet, indicating that eDNA associated rapidly with the Pse1 cells and that the proportion did not alter significantly with extended incubation times (Figure S3a). The binding of the eDNA was shown to be reversible as it was readily displaced by incubation with an excess (500 μg mL^–1^) of phage lambda DNA (Figure S3b). DNaseI treatment was much less effective at removing bound DNA, presumably because of the steric hindrance between the DNaseI enzyme and the relatively short DNA fragments used in this experiment.

**Figure 3:**
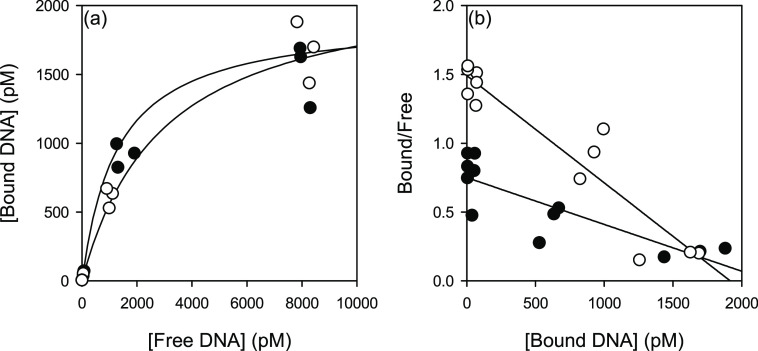
Binding of 750 bp DNA to PBS-washed (black) or DNaseI-treated (white) Pse1 cells. Binding data and a fitted line are shown as (a) free vs bound and the (b) Scatchard plot.

To determine the affinity of eDNA to the cell surface, ^33^P-labeled 688 bp DNA was incubated with PBS-washed and DNase-treated Pse1 cells at DNA concentrations between 10 and 5000 ng mL^–1^. Bound and free DNA were separated by centrifugation and quantified by scintillation counting. The binding characteristics of the labeled DNA to the cells are shown in Figure 3a, and the same data are shown in a linear form as a Scatchard plot (Figure 3b). The apparent affinity of eDNA to DNaseI-treated cells was 1.3 ± 0.1 nM, approximately half that of PBS-washed cells (2.9 ± 0.1 nM) due to competition with existing eDNA bound to the surface. Calculation of the maximum binding concentrations gave similar values (2.2 nM for PBS-washed cells and 1.9 nM for DNaseI-treated cells), corresponding to 13,250 and 11,433 DNA molecules per microbial cell. These results allow an estimation of cell coverage with eDNA. If the dimension of a *Pseudomonas* cell is considered to be a cylinder of 1 μm diameter and 2 μm length and the 688 bp DNA fragment to have dimensions of 2 × 234 nm, then one can calculate that more than 90% of the cell surface would be covered by eDNA, if evenly distributed.

### Influence of Ca^2+^ on Microbial Attachment

**Figure 4:**
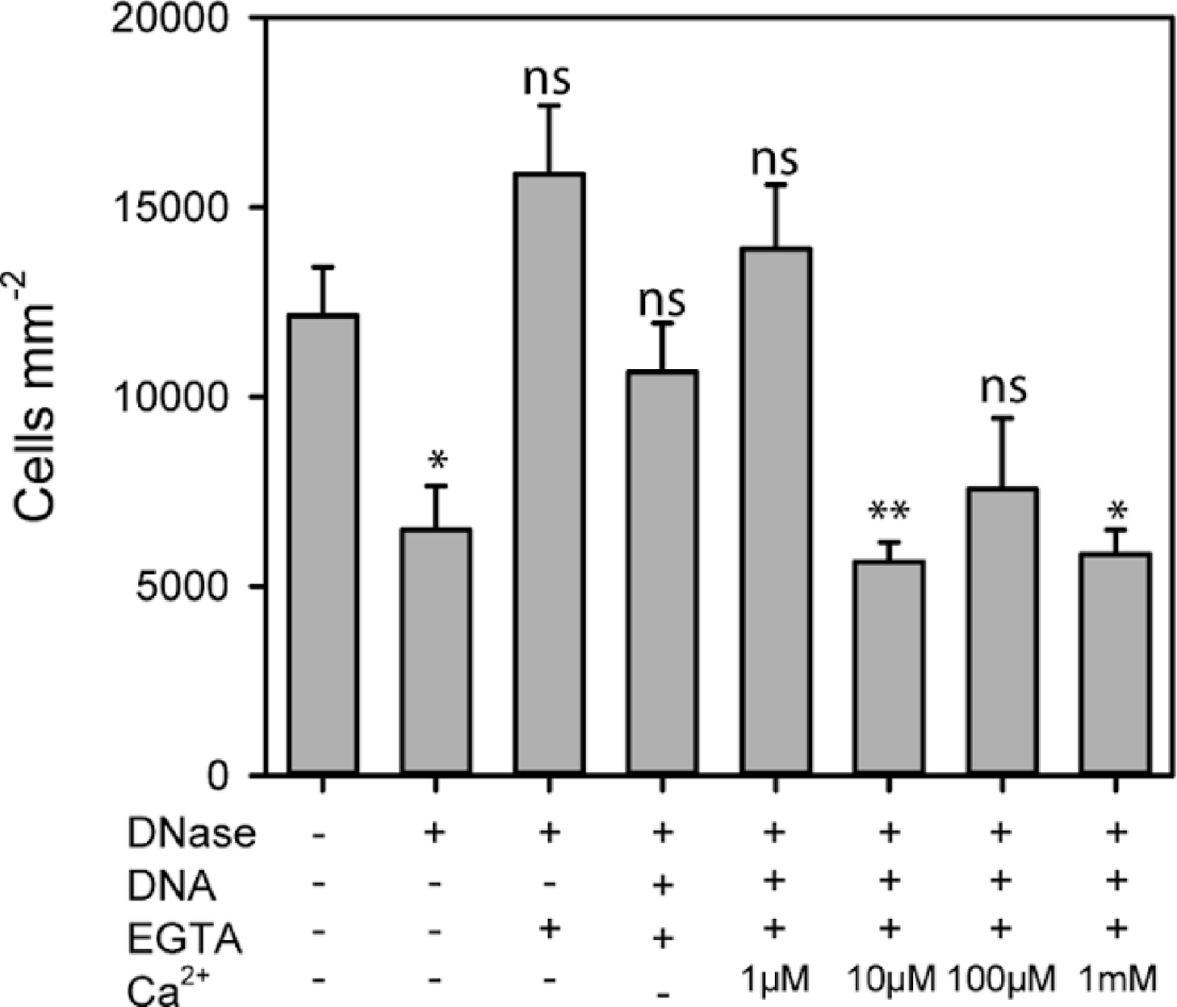
Cell attachment to fused silica surfaces under different assay conditions. Where indicated, cells were treated with DNaseI and then washed with EGTA to remove divalent cations. eDNA and Ca^2+^ were then readded to the assay medium. Results are means ± SE of three biological replicates. Statistically significant differences from the control are shown (*p < 0.05, **p < 0.01, and ***p < 0.001) (one-way ANOVA of log2-transformed data).

We investigated the role of Ca^2+^ in biofilm formation in the presence and absence of eDNA and chelating agents (Figure 4) because divalent cations can form bridges between negatively charged macromolecules on the microbial cell surface, eDNA, and the silica surface. Cell adherence was reduced by DNaseI treatment but increased by treatment with EGTA, a Ca^2+^ chelator. Readdition of eDNA restored binding, but surprisingly, attachment was reduced at Ca^2+^ concentrations of 10 μM and above, well within the physiological range expected in the environment.

### Influence of Cations on DNA Adhesion

**Figure 5:**
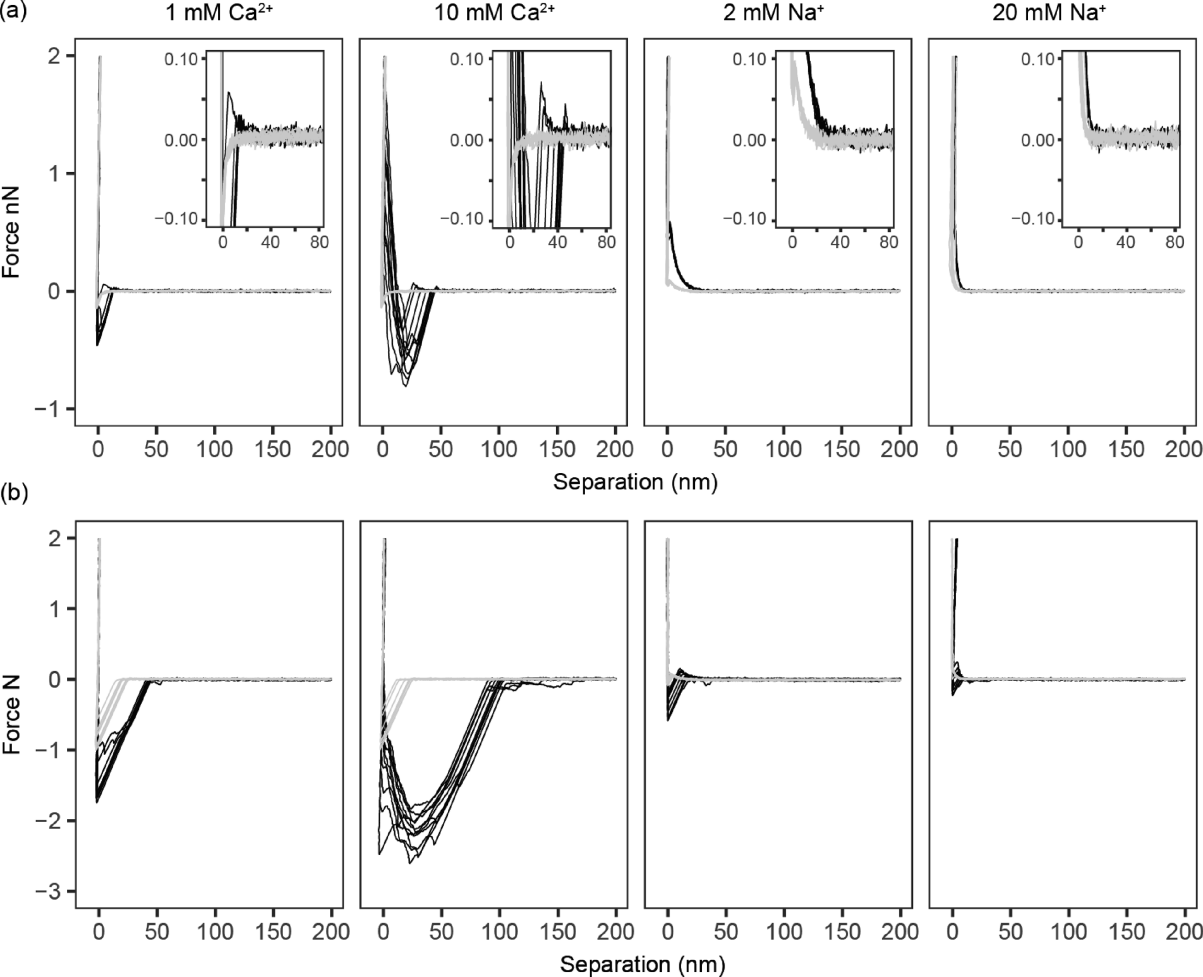
Approach (a) and retraction (b) curves for control (light gray) and DNA-coated (black) cantilevers interacting with a fused silica surface. Ten curves were selected at random from the complete data set.

To better understand the interaction between eDNA and surfaces, SMFS was used to probe the interactions between single DNA molecules and the native oxide surface of silicon wafers under a range of cationic conditions. DNA molecules were attached to the AFM tip and the interactions measured in 1 mM Ca^2+^, 10 mM Ca^2+^, 2 mM Na^+^, and 20 mM Na^+^ (the last is the Na^+^ concentration in PBS buffer). Extension and retraction curves were measured for all interactions, with a random selection of 10 interactions shown in Figure 5.

The strength of adhesion can be extracted from the total work needed to pull the DNA off the surface completely. To quantify these interactions, the areas below the retraction curves and the distance over which these interactions occurred were calculated (Figure S4 and Table S2).

For Ca^2+^, most of the AFM curves followed a similar pattern. Extension curves generally showed a small repulsive force (insets of Figure 5) followed immediately by a strong attraction (jump-to-contact) and then a constant compliance region. The repulsive force appears similar at both Ca^2+^ concentrations but occurred much further away in 10 mM Ca^2+^ (44.3 nm) than in 1 mM Ca^2+^ (14.6 nm). As the tip moved nearer to the surface, all curves measured in Ca^2+^ showed a jump-to-contact that also occurred at a greater distance from the surface with increasing Ca^2+^ concentration and in the presence of eDNA. The retraction curves measured in the presence of Ca^2+^ showed a dominant single large pull-off event, which would indicate an extended interaction resulting from the DNA lying flat on the silica surface. In some cases, extended interactions consisting of multiple smaller peaks were also observed that indicate multiple interactions between the DNA molecule and the surface. The size of the interaction was greater in 10 mM Ca^2+^ than in 1 mM Ca^2+^.

In contrast, with Na^+^, a large amount of variation in the approach curves was observed with some showing no binding. The most common case was when the AFM cantilever was repelled from the surface and slowly approached the constant compliance region without the jump-to-contact seen for Ca^2+^. The repulsive interaction decreased with increasing sodium concentration. (This was also the case for when no eDNA was attached to the AFM tip, but in that case, the interaction was much weaker. The results are included in Table S2.) For Na^+^, the retraction curves were varied with many showing no binding event. Those that showed binding generally demonstrated multiple peaks in the retraction curves with comparable sizes. The forces increased in the presence of eDNA and decreased with increased ionic strength, but again a large variation was observed across these. Overall, the interactions were far weaker in the presence of Na^+^ than Ca^2+^. For the sodium ions, the works done in detaching the AFM tip from the surface were 1 ± 3 aJ (2 mM) and 0.14 ± 1.5 aJ (20 mM), and for the calcium ions, the results were 34 ± 6 and 138 ± 17 aJ for 1 and 10 mM, respectively (Table S2).

The behavior of single DNA molecules was therefore in marked contrast to the observations of the whole cell behavior where the attachment was reduced at elevated Ca^2+^. To resolve this apparent contradiction, molecular dynamics simulations of DNA molecules interacting with silica surfaces were performed.

### Computational Simulations

**Figure 6:**
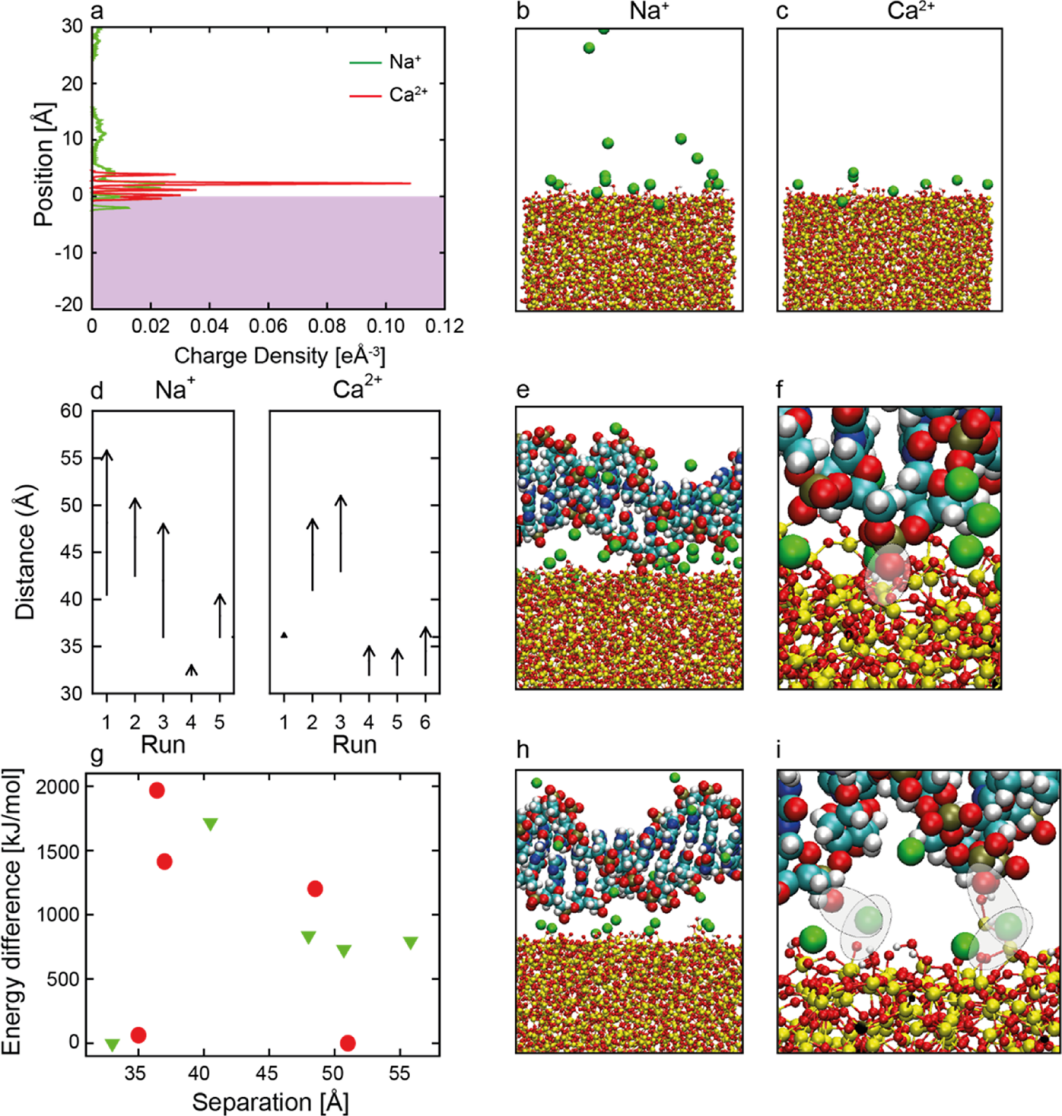
(a) Charge density of Na^+^ (green) and Ca^2+^ (red) cations traveling perpendicular to the silica surface (shown as a purple-shaded region) and aligned with the snapshot image simulations with (b) Na^+^ and (c) Ca^2+^ present. The cation is shown in green, O in red, H in white, and Si in yellow. Water molecules have been omitted for clarity. (d) Perpendicular distance between the center of mass of the amorphous silica slab and the DNA molecule and the surface of the amorphous silica slab at the beginning and end of the simulations. Snapshots of simulations of a DNA molecule binding to the silica surface in the presence of Na^+^ (e, f) and Ca^2+^ (h, i). Colors are the same as panels (b, c) with C (light blue), N (dark blue), and P (gold). The final energies of the simulation (g) with respect to the lowest energy simulation for the relevant cation system (Na: green triangles, Ca: red circles).

Computational simulations were performed for an amorphous silica surface in the presence of Na^+^ or Ca^2+^ ions in solution, with and without DNA present. Figure 6a shows the charge density (related to the probability of finding an atom) of different atoms in the plane perpendicular to the silicon surface in the absence of DNA. The Si atoms of the slab (shown as the dotted blue line) terminate at ∼4 Å. The Ca^2+^ ions form an organized space charge layer ∼2.2 Å from the slab surface. This is a basic feature of space charge layers produced by doubly charged ions. On the other hand, the singly charged ion Na^+^ produces a diffuse space charge layer over a wider range of ∼1–3.5 Å. Snapshots of Na^+^ and Ca^2+^ ions interacting with the surface are shown in Figure 6b and Figure 6c, respectively.

Figure 6d shows the perpendicular distance, *z* between the silica surface, and the center of mass of the DNA chain for a series of simulations. When Na^+^ was present, the DNA chain usually drifted away from the surface and ended the simulation further from the surface than its starting location. DNA binding to the surface was observed in only one simulation, where the starting point of the DNA molecule was close to the surface. (SMFS experiments also showed examples in the presence of sodium where binding did not occur.) This implies that the attractive interactions between the surface and DNA chain were relatively short-ranged and that an energy barrier to DNA binding was present. The bound configuration is shown in Figure 6e,f. The main interactions between the silica and DNA were direct interactions between the phosphate oxygen in the DNA and a silicon atom on the surface, as observed previously for DNA-silica.^39^

When Ca^2+^ was present, the DNA tended to move toward a center-of-mass separation of 3.6 nm when the simulation was started with the molecule relatively close to the surface, indicating that this distance was energetically preferred. When simulations were started with the DNA further away, it tended to drift away from the surface, indicating that longer-range attractive forces were not present. The bound configuration is shown in Figure 6h. The Ca^2+^ ions were still organized in a tight space charge layer, and all interactions between the DNA and the surface were mediated by Ca^2+^ ions, as highlighted in Figure 6i.

The role of the cations in mediating interactions between the DNA molecule and the surface was investigated by looking for simultaneous complexation of the cation to both the surface and DNA. Table 1 shows the number of oxygen (phosphate)–cation–oxygen (silica) complexes present during the simulations and their lifetimes. Few oxygen (phosphate)–Na^+^–oxygen (silica) complexes were present, and very few had a lifetime longer than 500 ps, indicating that, although these complexes may have contributed to the interactions, they were not a dominant component of the energetics. The small charge of Na^+^ resulted in weak solvent shells in solution and correspondingly weak complexation forces. On the other hand, many long-lived bridges of the type oxygen (phosphate)–Ca^2+^–oxygen (silica) were present, including some within the first solvent shell. This demonstrates the role of Ca^2+^ ions in mediating DNA–surface interactions. This much more structured and tighter binding arrangement between DNA and the silica surface with calcium present agrees with the enhanced stiffness observed for calcium compared to sodium in QCM studies of silica–DNA systems.^31,32^ The more rigid structure of the silica–Ca–DNA system should more directly affect any vibrations than the more flexible silica–Na–DNA systems.

**Table 1:** Number of Inner/Outer Shell O–Na–O and O–Ca–O Complexes Present in the Simulations^a^

solvation shell interactions	1st–1st	1st–2nd	2nd–1st	2nd–2nd
Na^+^	1/0/0	18/1/1	18/5/0	149/24/12
Ca^2+^	1/1/1	12/7/5	8/8/7	61/40/26

a Values are listed for 50, 200, and 500 ps. The column headings refer to the solvation shell of the cation in which the O atom is located.

Figure 6g shows the final configurational energy of each simulation with respect to the lowest energy simulation of each cation system. In the presence of Na^+^, the energy is approximately +800 kJ mol^–1^ when the DNA is far from the surface. As the molecule approaches the surface, the energy rises to approximately +1700 kJ mol^–1^, indicating an energy barrier of ∼900 kJ mol^–1^. It then reduces to zero as the DNA molecule binds to the surface. Therefore, binding with Na^+^ present is thermodynamically favored by ∼800 kJ mol^–1^ (69 mJ m^–2^). These large values of binding energy relate to the interactions of many atoms (an infinite DNA chain with an 11 base repeat), and there is a substantial Coulombic charge between the systems. With Ca^2+^, the situation is quite different. When DNA is far from the surface, it has a low energy that is numerically comparable to the energy of the simulation when the DNA is closer to the surface at the preferred 3.6 nm separation. Hence, there is no configurational energetic preference for the DNA to be bound at the surface rather than “free” in solution. These are configurational energies and do not include an entropic component. The displacement of water and changes in solvation of the cations during the binding processes would be the main contributors to changes in the entropy. Given the generally small changes that happen in the Ca^2+^ system (i.e., the solvation of the Ca^2+^ layer is largely maintained, and the surface water on the silica is not displaced), the entropy change in this binding may not be too significant. For Na^+^, this is harder to estimate due to the changes taking place in the Na and surface solvation.

## Discussion

We have investigated the role of eDNA in the adherence of *Pseudomonas* Pse1 to silica surfaces. This strain produced 60 ng mL^–1^ eDNA in the early stationary phase. The timing and amount of eDNA production vary widely between different bacteria, but this value is similar to that reported for *S. mutans* (85 ng mL^–1^ over 10 h).^54^ It was found that cell attachment was strongly influenced by eDNA but not eliminated entirely by DNaseI, irrespective of the time of treatment. DNaseI treatment reduced cell attachment and cell aggregate formation, indicating a role for eDNA in both cell–surface and cell–cell interactions.

The computational simulations show that the negatively charged DNA interacts with the surface through the positively charged Ca^2+^ Stern layer above the negatively charged silica surface. Such models have been reported for a similar system of mica–DNA interactions with divalent cations present.^33^ This generally uniform interaction is consistent with the polymer adsorbed to the surface with few “loops” into the medium. As the Ca^2+^ concentration is increased, this layer becomes more complete across the surface leading to a more substantial interaction, which is again borne out by the SMFS experiments. This interaction is expected to be relatively long-ranged as shown by the pulling of the DNA toward the surface in all the simulations where the initial center-of-mass separation between the DNA and the silica surface was 3.6 nm. This is supported by the jump-to-contact behavior and long-range retraction curves shown in the force spectroscopy experiments.

**Figure 7:**
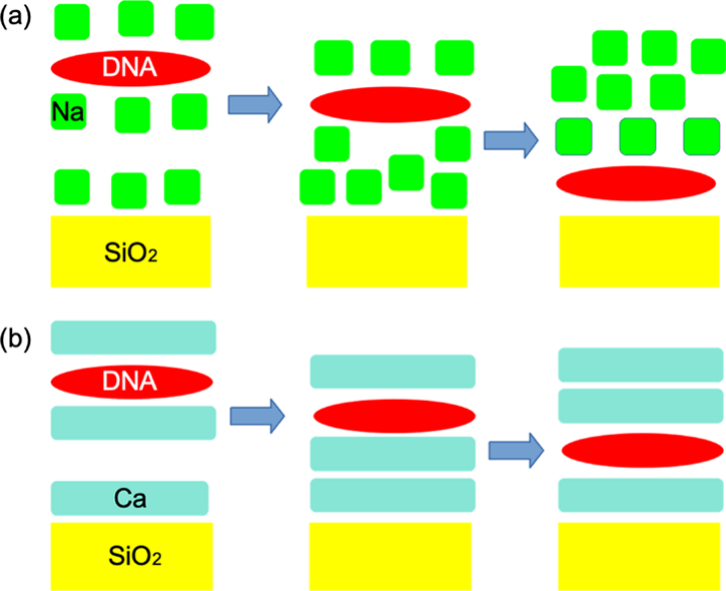
Schematic diagram illustrating the barriers that need to be overcome for the DNA to adsorb on the surface. In the sodium case (a), the DNA can move through a disorganized Na^+^ layer and can reach the SiO_2_ generating new favorable interactions. For calcium (b), the DNA must push through an organized Ca^2+^ layer, which is difficult. It cannot fully reach the surface and therefore generates no new favorable interaction.

Microscopic measurements of the whole cell behavior demonstrated the importance of eDNA in attachment.^55^ It was also found that cell attachment was reduced at high Ca^2+^ concentrations. However, the SMFS measurements presented here show that high Ca^2+^ concentrations strengthen the interaction between single DNA molecules and the substratum. This means that the binding between eDNA and the surface is reduced by screening (see the schematic diagram in Figure 7). However, if the eDNA is close enough to the surface, then screening plays less of a role, and the binding is strong. This can be seen from the computer simulations, which demonstrate that a large, wide barrier exists for the removal of the DNA from the surface as we pull the negative DNA away from the positive Ca^2+^ layer on the surface, in agreement with the retraction curves. There also exists a substantial energy barrier to bringing the DNA into its bound state near the surface. The DNA in solution is strongly complexed by Ca^2+^ cations. The silica surface also has a high concentration of bound Ca^2+^, forming a layer. As the DNA moves toward the surface, these two positive Ca^2+^ layers are forced toward each other and eventually merge. This process must produce a large Coulombic repulsion leading to the large barrier as observed in the simulations and the AFM approach curves.

In the Na^+^ system, there is no organized Na^+^ layer either above the surface or around the DNA so the eDNA adsorption behavior is different from that of calcium ions. First, the lack of an organized Na^+^ structure means that the distribution of the Na^+^ ions would vary across the sample and between experiments, which relates to the larger variation observed in the AFM curves for the Na^+^ cases. Interactions between the surface and the DNA are now close-ranged and between specific atoms. This leads to atomic binding events rather than the more generic DNA chain–surface interaction seen in the Ca^2+^ system. The SMFS experiments are consistent with this, showing a greater frequency of retraction profiles exhibiting multiple events in the presence of Na^+^ compared to Ca^2+^, which can be related to breaking of individual binding points along the DNA during retraction. Furthermore, many retraction curves were observed where no clear interaction between the tip and DNA was present, which would correspond to a failure to form specific interactions. The binding in the SMFS experiments in the presence of Na^+^ was weaker than that in Ca^2+^ solution. This corresponds to the smaller barriers calculated for the Na^+^ system. The simulations showed that binding leads to an overall decrease in the system energy as we generate new interactions between the DNA and surface. This relates to the binding seen in the cellular systems with only Na^+^ present.

Although the kinetics of single-stranded DNA adsorption in the presence of divalent magnesium ions to (hydroxylated) silica surfaces has been seen to be significantly slower than for sodium ions, which is consistent with the energy barrier,^56^ other experiments have shown that DNA adsorbs much more rapidly in the presence of calcium ions than sodium.^32^ Ultimately, kinetics experiments are not an effective probe of the presence of an energy barrier because the adsorption rate of DNA is a function of concentration, which is optimized for the experiment in question.^31^

The computational simulations also showed that there was little difference between the energies of bound DNA and free DNA in solution. In both cases, the silica surface and the DNA are solvated by organized Ca^2+^ layers, and therefore, the energy would not be expected to be significantly different. This creates a substantial energy barrier preventing binding of the DNA but only a weak thermodynamic driving force for binding (as the process does not significantly lower the energy of the whole system). Hence, in the presence of Ca^2+^ ions, DNA shows a weak thermodynamic binding to the surface but can be strongly kinetically trapped there. This explains why the force spectroscopy data show such strong attachment, but the Pse1 exhibits reduced binding in the presence of calcium (Figure 4).

In the SMFS experiments, an increase in the concentration of Na^+^ from 2 to 20 mM led to a decrease in the average strength of binding in the retraction curves. An increase in the concentration of Na^+^ in the solution increases the concentration of Na^+^ both at the surface and around the DNA. This will not lead to the double-layer binding observed for Ca^2+^ ions but, depending on the localized concentration and geometry, could lead to regions of Na^+^ on the surface and around the DNA being forced together. An increased concentration of sodium ions therefore reduces eDNA binding by disrupting the formation of individual binding events, creating the repulsion between the DNA and surface observed in the SMFS experiments. The simulations could not capture this process since the concentrations are fixed and relatively evenly distributed around the molecule and surface over a small length scale. The very large (microscale) simulations that would be required to generate the variation in ion distribution are beyond the scope of this study.

A direct comparison between eDNA binding energies to silica obtained from molecular dynamics simulations and SMFS experiments is beyond the scope of this work. Dynamic force spectroscopy is needed to obtain experimental energy barriers, whereby the potential barrier varies with loading rate.^57^ Typically, molecular dynamics simulations, which require very small time steps, correspond to loading rates significantly greater than the 40 nN s^–1^ used in these experiments,^58^ which further complicates any comparison.

## Conclusions

Taken together, single-molecule force spectroscopy and molecular dynamics simulations allow an understanding of the confusing picture of cellular adhesion to silica surfaces. When only Na^+^ is present, a favorable interaction of the eDNA with the surface and significant cellular attachment are both observed. This is due to thermodynamic binding. Adding Ca^2+^ ions will replace the singly charged Na^+^ ions on the silica surface and around any eDNA molecule. There will then be no energy gain for binding eDNA to the surface from the solution. The presence of Ca^2+^ in the solution will therefore inhibit cellular adhesion. In the AFM studies, the eDNA is held tightly to the surface because there is a large barrier to its detachment (since this must disrupt the Ca^2+^ layer). This explains the large work of adhesion observed in these studies.

Other studies have suggested that binding should be stronger in calcium-based systems using arguments based on enhanced adsorption rates in isothermal approaches.^32^ That method examines saturation at the surface and therefore is concerned with much greater concentrations of DNA than we consider. The enhanced binding rate may be a result of better charge screening by the divalent Ca^2+^ cations allowing DNA molecules to cluster together.^59^ This will let DNA molecules accumulate on the surface in far greater numbers than is possible with the (less effective) monovalent Na^+^. Furthermore, there will also be a steric hindrance between relatively free single DNA molecules on an AFM tip or in the molecular dynamics simulations and those on bacterial surfaces.

A mechanistic understanding of biofilm formation and cell adhesion to surfaces requires approaches that span a huge range of physical scales, from atomic interactions between macromolecules and substrata to the behavior of populations of cells. Here, different methodologies have been combined to develop an integrated model of cell attachment, identifying the interactions that facilitate adhesion by eDNA and relating this to the bulk cell behavior. This integrated approach has enabled the resolution of apparent contradictions that arise from different approaches and thus deepened our understanding of biofilm formation.

## Supporting Information Available

Molecular dynamics simulation run, cell adherence data, DNA binding data, force spectroscopy histograms, parameters used for molecular dynamics simulations, and force spectroscopy results (PDF)
