# Supplementary material for: The Role of Extracellular DNA in Microbial Attachment to Oxidized Silicon Surfaces in the Presence of Ca2+ and Na+
Source: Langmuir. 2021 Aug 4;37(32):9838–50. doi: 10.1021/acs.langmuir.1c01410 (PMC8397393; doi:10.1021/acs.langmuir.1c01410)
Supplement: Supplementary file 1 — la1c01410_si_001.pdf [file la1c01410_si_001.pdf]

Supporting Information

The role of extracellular DNA in microbial attachment to oxidized silicon surfaces in the presence of Ca^{2+} and Na^{+}

Ana L. Morales-García,[#] Rachel Walton, [‡,#] Jamie T. Blakeman,[#] Steven A. Banwart,[ø] John H. Harding,[†] Mark Geoghegan,[#] Colin L. Freeman,[†] and Stephen A. Rolfe[‡]

Department of Physics and Astronomy, The University of Sheffield, Hounsfield Road, Sheffield S3 7RH, UK

‡ Department of Animal and Plant Sciences, The University of Sheffield, Western Bank, Sheffield S10 2TN, UK

ø Department of Civil and Structural Engineering, The University of Sheffield, Sheffield S3 7HQ, UK

S2 Figure S1. Molecular dynamics simulation run
S3 Figure S2. Cell adherence data
S4 Figure S3. DNA binding data
S5 Figure S4. Force spectroscopy histograms

S6 Table S1. Parameters used for molecular dynamics simulations
S7 Table S2. Force spectroscopy results

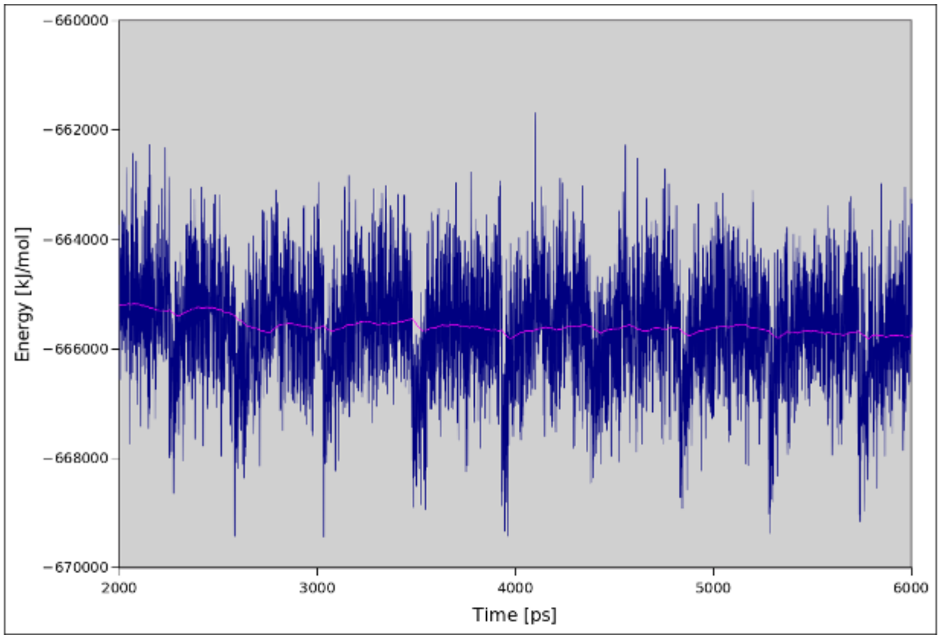

Figure S1. Energy from a simulation run covering a 2 ns to 6 ns region. The final 1 ns is the production part where structural data are collected. The data show that the energy has reached equilibration and that the moving average (pink line) is reliable.

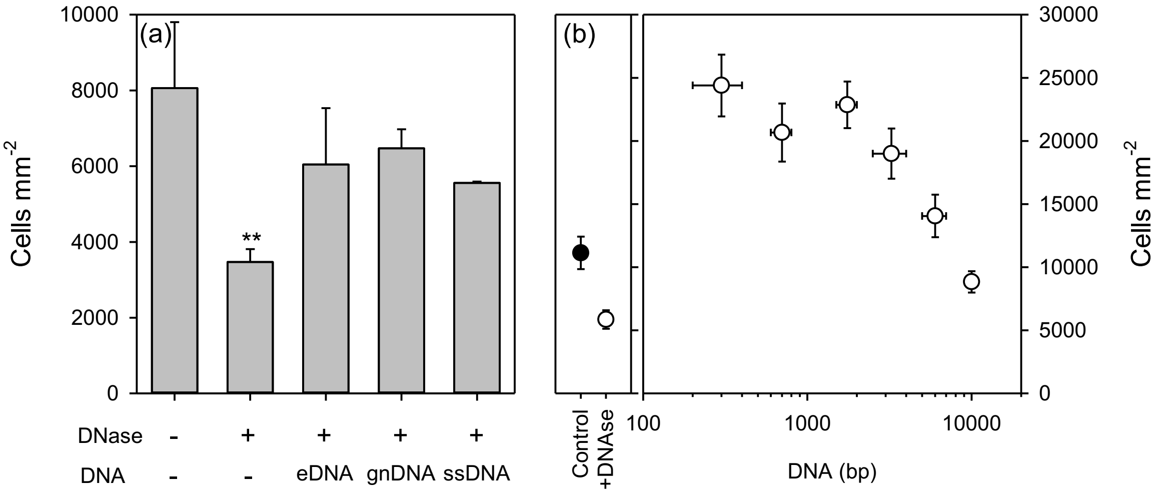

Figure S2. (a) Extracellular DNA (eDNA) isolated from the growth medium, Pse1 genomic DNA (gnDNA) and salmon sperm DNA (ssDNA) were all equally effective at restoring binding of DNaseI-treated cells to fused silica slides. Values are means ± SE (n = 3). ** means value is significantly different from the control (One-way ANOVA). (b) Binding of DNase-treated cells was enhanced by the addition of ssDNA of different sizes. Results for binding are the means ± SE (n = 3), the horizontal bars show the size ranges of the DNA fragments. Control (PBS-washed cells, filled circles) and DNase-treated cells without eDNA additions are shown for comparison.

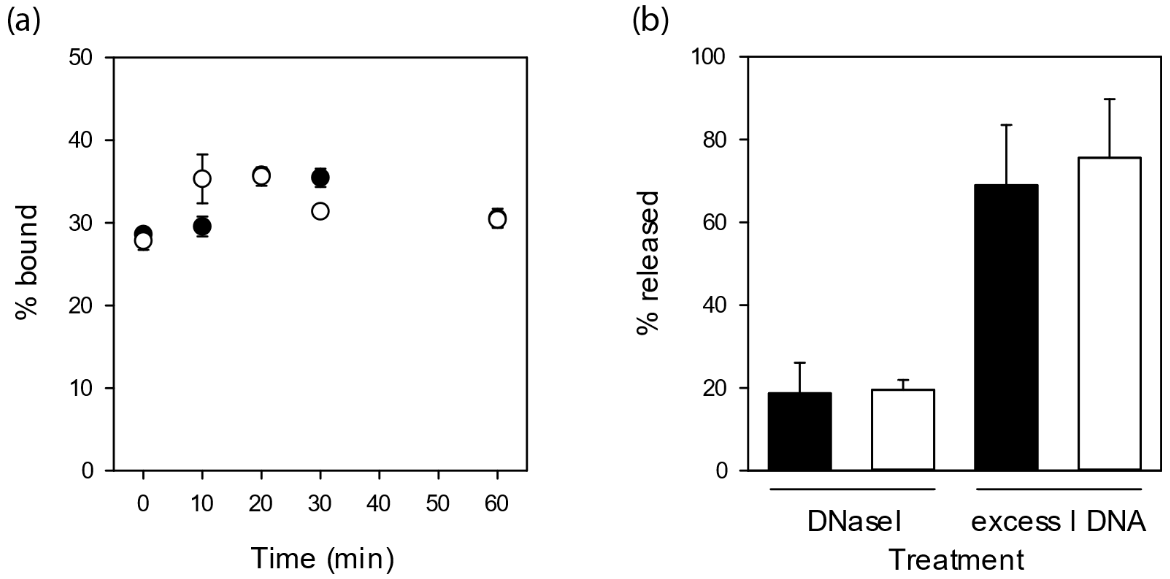

Figure S3. (a) Kinetics of 700 bp DNA binding to Pse1 cells. Pse1 cells were incubated with ^{33}P-labelled 700 bp DNA and the proportion bound determined at intervals. Results are the means ± SE (n = 3) for PBS-washed (white) and DNaseI-treated (closed symbols) cells. (b) Release of 700 bp DNA from the surface of PBS-washed (black) or DNaseI-treated (white) Pse1 cells by treatment with DNaseI or 500 ng ml^{-1} phage λDNA. Values are means ± SE (n = 3)

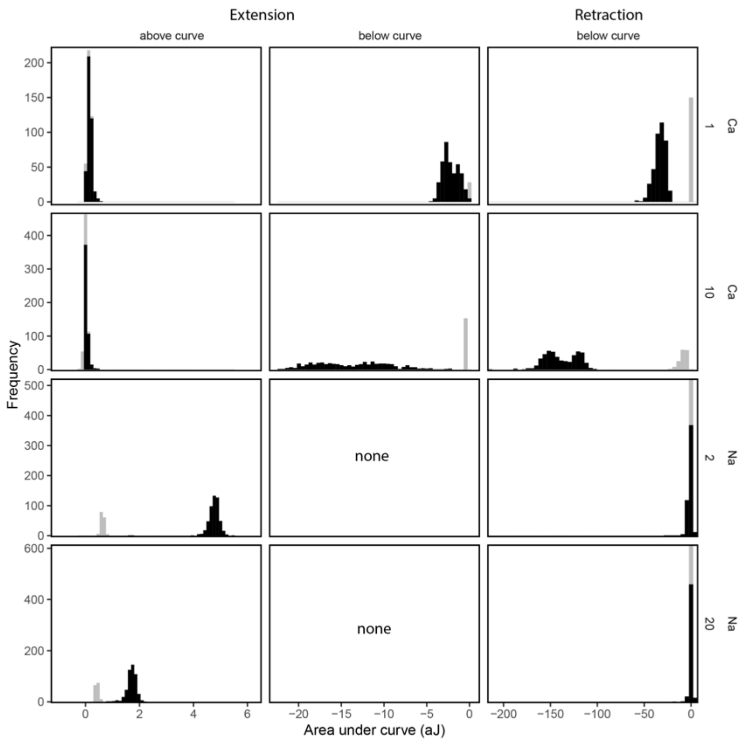

Figure S4. Histograms of the work done for the eDNA-functionalized AFM tip approaching the surface and retracting from it in the presence of calcium (1 and 10 mM) or sodium (2 and 20 mM) cations. Mean data values are tabulated in **Table S2**. Black and gray bars show values from AFM tips with and without eDNA respectively

Atoms			Type	k (eV mol^{-1})	θ (°)
Si	O	H	harmonic	12.0	115.7165

Table 1a. Parameters used in the Si-O-H harmonic angular interaction.

Atoms		A (eV)	ρ (Å)	C (eV Å^6)
Si	O (phosphate single bonded O – OS)	15140.0	0.20520	133.54
Si	O (phosphate double bonded O)	16212.0	0.20520	133.54
Si	N	15468.0	0.20520	133.54
Ca	O (silica)	827451.3	0.279654	103.07
Na	O (silica)	5151.6	0.272500	60.0

Table S1b. Parameters used in Buckingham intermolecular interactions.

Atoms		A (eV Å^{12})	B (eV Å^6)
O (silica)	P	259716.9	117.71

Table S1c. Parameters used in A-B intermolecular interactions.

Condition	Approach				Retraction	
	Distance (nm)	Work done (aJ)	Distance (nm)	Jump-to-contact (aJ)	Distance (nm)	Total work done (aJ)
1 mM Ca^{2+}	-	-	1.57 ± 0.15	–0.007 ± 0.001	6.8 ± 2.4	–0.5 ± 0.3
1 mM Ca^{2+} (eDNA)	14.6 ± 0.1 (46%)	0.11 ± 0.02	11.0 ± 0.3	–2.18 ± 0.05	47 ± 11	–34 ± 6
10 mM Ca^{2+}	-	-	13.2 ± 0.3	–0.446 ± 0.005	22 ± 5	–8.4 ± 3.9
10mM Ca^{2+} (eDNA)	44.3 ± 0.8 (30%)	0.11 ± 0.01	41.6 ± 0.2	–13.6 ± 0.2	109 ± 23	–138 ± 17
2 mM Na^{+}	11.8 ± 0.2	0.636 ± 0.005	-	-	13 ± 2	0.65 ± 0.27
2 mM Na^{+} (eDNA)	23.8 ± 0.1	4.75 ± 0.01	-	-	26 ± 9	–1.18 ± 3.35
20 mM Na^{+}	4.1 ± 0.1	0.421 ± 0.005	-	-	5.7 ± 0.8	0.5 ± 0.1
20 mM Na^{+} (eDNA)	8.2 ± 0.03	1.70 ± 0.01	-	-	16 ± 13	–0.14 ± 1.5

Table 2. Quantification of interactions of AFM tips with fused silicon oxide surfaces. Measurements were made with and without eDNA under different ionic conditions. The work done is simply the area under (or over) the force-distance curve. For approach curves, the work done is that by the AFM on the surface, so that jump-to-contact values are negative. For retraction curves, positive values indicate that the tip is rejected by the surface, so that work is done *on* the AFM tip. Values are means ± standard deviations. Distance is the separation distance at which the interaction first (extension) or last (retraction) occurred. Values in brackets indicate the number of curves where the interaction was seen if this was less than 100%.
